# Epilepsia partialis continua in mitochondrial dysfunction: Interesting phenotypic and MRI observations

**DOI:** 10.4103/0972-2327.42942

**Published:** 2008

**Authors:** Kalyani Karkare, Sanjib Sinha, Shivashankar Ravishankar, Narayanappa Gayathri, T. Chikkabasavaiah Yasha, Manoj K. Goyal, Joy Vijayan, Ayyasamy Vanniarajan, Kumarswamy Thangaraj, Arun B. Taly

**Affiliations:** Department of Neurology, NIMHANS, Bangalore, India; 1 Department of Neuroimaging and Interventional Radiology, NIMHANS, Bangalore, India; 2 Department of Neuropathology, NIMHANS, Bangalore, India; 3 Centre for cellular and molecular Biology, Hyderabad, India

**Keywords:** Chronic progressive external ophtalmoplegia, epilepsia partialis continua, mitochondrial dysfunction, Mitochondrial Encephalopathy with Ragged Red Fiber, MRI, periodic lateralized epileptiform discharges

## Abstract

An 11-year-old girl manifested with photophobia, ptosis, external ophthalmoplegia, hypotonia, weakness of proximal limb muscles, hyporeflexia, and generalized seizures (six months). Her elder sister had had uncontrolled seizures and photophobia and died at seven years of age. In the patient, serum lactate was high (55 mg/dl). Muscle biopsy revealed characteristic ragged red and ragged blue fibers, diagnostic of mitochondrial cytopathy. Sequencing of the complete mitochondrial genome of the DNA obtained from the muscle biopsy of the patient did not show any characteristic mutation. Four months later, the girl was admitted with a one-week history of epilepsia partialis continua (EPC). EEG revealed Periodic Lateralized Epileptiform Discharges (PLEDs), once in 2-4 seconds, over the right temporo-occipital leads. MRI revealed signal change of right motor cortex, which had restricted diffusion. MR spectroscopy (MRS) from this region revealed lactate peak. EPC remained refractory to multiple anti-epileptic drugs, immuno-modulators, coenzyme-Q, and carnitine. This thought provoking report expands the spectrum of mitochondrial cytopathies.

## Introduction

Epilepsia partialis continua (EPC) has varied etiologies. In children, it is attributed to birth related injury, neuroinfection and Rasmussen's encephalitis.[1,2] To a large extent, its treatment depends on the underlying etiology, in addition to the prompt use of anti-epileptic drugs (AEDs). Mitochondrial cytopathy, a multisystem disorder, also involves the nervous system.[3,4] The epileptic phenotypes in these disorders are mostly generalized seizures, partial motor seizures and myoclonus. While most of the literature regarding this is limited to mitochondrial encephalopathy, lactic acidosis and stroke-like episodes (MELAS), there are only a few reports of Epilepsia Partialis Continua (EPC) in patients with Mitochondrial Encephalopathy with Ragged Red Fiber (MERRF), Chronic Progressive External Ophtalmoplegia (CPEO) etc.[5]

We report a patient of mitochondrial cytopathy, who manifested with epilepsia partialis continua, and propose a pathogenetic link.

## Case Report

**Figure 1:**
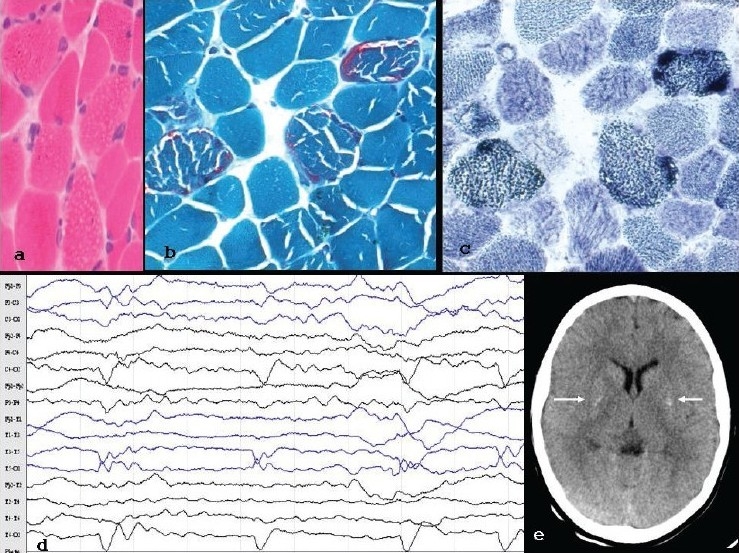
(a-c) Biopsy of biceps showing ragged appearing fibers on Haematoxylin and Eosin stain (a) which on MGT (b) and SDH (c) are ragged red and ragged blue respectively d) EEG with Periodic Lateralised Epileptiform Discharges (PLEDs) - once in 2-4 seconds, over right>left temporo-occipital leads e) Axial CT scan with bilateral basal ganglia calcification

An 11-year-old girl, the younger of two siblings, born to nonconsanguineous parents, was initially evaluated for photophobia of five years, drooping of eyelids of two years and recurrent generalized tonic clonic seizures of six months' duration. Her elder sister had photophobia and seizures from the age of five; she died at seven years of age, due to possible complication of uncontrolled seizures. She was not evaluated at our center and the medical reports were not available. The parents were evaluated by us; they did not have any history of similar illness. The patient had bilateral ptosis, complete ophthalmoplegia with sparing of pupil, hypotonia, weakness of proximal muscles of lower extremities (MRC: 4+/5), and global hyporeflexia. There was no other deficit. The following laboratory tests revealed normal results: urine analysis, complete hemogram, serum chemistry including glucose, renal and hepatic function tests, electrolytes and creatine phospokinase (CPK). Serum lactate level was 55 mg% (Normal: 4.5-20). Blood gas analysis, including pH, was normal. Cerebrospinal fluid (CSF) lactate and serum pyruvate analysis were not carried out. The nerve conduction parameters in the right median (motor and sensory), common peroneal and sural nerves, and the electromyographic studies in biceps and quadriceps were normal. Cranial CT scan showed bilateral basal ganglia calcification. [Figure 1E]. CSF was acellular, with glucose of 60 mg% and protein of 45 mg%. A diagnostic muscle (biceps) biopsy revealed characteristic ragged red fibers on Modified Gomori's Trichrome (MGT) and ragged blue fibers on succinate dehydrogenase (SDH) stain. [Figures 1A–C]. The complete mitochondrial genome (16569 base pairs) obtained from the muscle biopsy of the patient was sequenced and compared with the sequences with revised Cambridge Reference Sequence (rCRS) and normal age matched controls. A total of 29 variations were observed in the patient. All of them were either polymorphic or silent mutations. There was no characteristic mutation accountable for the pathogenesis. Measurement of the enzymatic activity of respiratory chain (RC) complexes and pyruvate dehydrogenase complex (PDH) could not be carried out, due to lack of facility.

**Figure 2:**
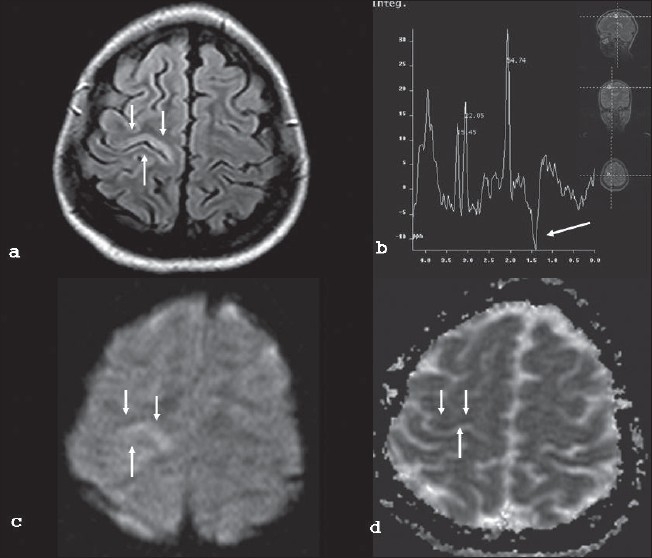
(a) Axial MRI (FLAIR) showing hyperintense right motor cortex b) MR Spectroscopy showing a lactate peak at 1.4 c, d) Diffusion weighted image (DWI) revealing hyperintensity in the right motor cortex which was hypointense on apparent diffusion coefficient (ADC) suggesting restricted diffusion

Four months later, the patient manifested with uncontrolled epilepsia partialis continua, involving the left upper and lower limb, of six days' duration. The routine EEG showed background in the theta range, with Periodic Lateralized Epileptiform Discharges (PLEDs), recurring once in 2-4 seconds, over right>left temporo-occipital leads [Figure 1D]. The MRI of brain in a 1.5 Tesla system revealed focal area of hyperintense signal intensity changes over the right motor cortex on FLAIR and Diffusion Weighted Image (DWI), which became hypointense on apparent diffusion co-efficient (ADC) mapping, suggesting restricted diffusion. MR spectroscopy (MRS) of the involved region revealed a lactate peak at 1.4 of the spectra. [Figures 2A–D].

A diagnosis of mitochondrial cytopathy, possibly CPEO plus syndrome or MELAS was considered. She received multiple anti-epileptic medications (phenytoin, phenobarbitone, clobazam, valproate, and carbamazepine), immunomodulators (methylprednisolone, intravenous immunoglobulin) and other agents like multivitamins and co-enzyme Q, but the EPC remained poorly controlled for the next month. She was lost to follow-up subsequently.

## Discussion

Mitochondrial cytopathy has protean manifestations: cataract, short stature, retinitis pigmentosa, heart block, lipoma, GI dysfunction, myopathy, neuropathy, deafness, cognitive decline, ataxia, involuntary movements like dystonia, and respiratory distress, among others.[3,4] The neuroimaging observations are varied and include symmetrical basal ganglia calcifications, signal alterations in putamen, thalamus, midbrain, dentate nuclei, multifocal small ischemic zones of varying ages, which do not respect the known arterial territory, and elevated lactate peak in MRS spectra.[5]

Our patient had characteristic phenotype, elevated serum lactate, basal ganglionic calcification, detectable lactate peak on MRS, and pathognomonic histo-pathological features of mitochondrial cytopathy. She developed refractory epilepsia partialis continua, with the EEG showing PLEDs and the MRI revealing focal cytotoxic edema. This association is rarely reported, though there are a few studies of seizures in mitochondrial disorders.[5–8]

Hori *et al.*[6] studied patients with mitochondrial encephalopathy, lactic acidosis and stroke-like episodes (MELAS) and seizures and reported two types of seizures: generalized and partial. These patients frequently manifested with visual symptoms and hemiparesis and might have posteriorly dominant EEG abnormalities. Veggiotti *et al.*[7] had reported uncontrolled EPC in a patient of MELAS. Feddersen *et al.*[8] described an interesting manifestation of nonconvulsive status epilepticus manifesting as aggressive confusional state in a patient with MELAS. Similarly, complex partial status epilepticus and EEG revealing PLEDs in patients with MELAS have been described.[9,10] Epilepsia partialis continua was reported by Stenqvist *et al.*[11] occurring two months preterminally in a six-year-old child with MELAS. Ribacoba *et al.*[12] described their observations on four adult (27-41 years) patients of MELAS manifesting with EPC. The seizures in these patients were precipitated by fever, headache and diabetic ketoacidosis.

MR spectroscopy in our patient had revealed a lactate peak, consistent with the diagnosis of mitochondrial dysfunction. However, lactate peaks over epileptiform foci were reported in a group of patients with tuberous sclerosis, without mitochondrial abnormalities.[13] More interestingly, the patient had signal alteration over corresponding motor cortex, suggesting cytotoxic edema due to either ischemia or local dysmetabolism. Kim *et al.*[14] have reported reversible cytotoxic edema on diffusion-weighted images (DWI) and restoration of NAA levels in a patient of MELAS and status epilepticus. We propose that local metabolic dysregulation could have caused seizure in our patient, similar to the observation made by Ribacoba *et al.*[12] Any cellular stress in a patient with mitochondrial cytopathies might be precipitated by hypermetabolic conditions like fever, hypoglycemia, and hypoxemia, which in turn could cause mitochondrial dysfunction manifesting as either neurological deficit or seizures.

Metabolic dysfunction as a cause of stroke in MELAS has been proposed by some studies, as has been revealed by the sensitive single photon emission computed tomography (SPECT) scan.[15] A similar mechanism could be extrapolated in the pathogenesis of seizures. Studies with newer sequences of MRI could unfold the underlying pathology in mitochondrial disorders like MELAS. Alternatively, the diffusion changes in MRI could be due to continuous seizures *per se*; however, serial MRI study could not be carried out to substantiate it.

Periodic Lateralised Epileptiform Discharges or PLEDs are EEG abnormalities characterized by repetitive spike or sharp wave complexes, recurring at variable intervals of 0.5-3 s. Pohlmann-Eden *et al.*[16] considered PLEDs as an EEG signature of a dynamic pathophysiological state, due to unstable neurobiological process creating an ictal-interictal continuum. The nature of the neuronal injury, preexisting propensity to have seizures, and the co-existence of dysmetabolic state, like in our patient, might contribute to seizures. The periodic discharges were recorded when her seizures were uncontrolled and she was on multiple AEDs. The occurrence of PLEDs in the presence of such a focal lesion might be a reflection of heightened neuronal excitability.

The drawbacks of this report are: lack of measurement of the enzymatic activity of respiratory chain (RC) complexes and pyruvate dehydrogenase complex (PDH), and inability to carry out serial MRI study due to lack of follow-up. Nevertheless, reporting of such patients could further expand the spectrum of mitochondrial diseases and unravel the underlying pathology by noninvasive means.

## References

[1] Cockerell OC, Rothwell J, Thompson PD, Marsden CD, Shorvon SD (1996). Clinical and physiological features of Epilepsia partialis continua. Cases ascertained in the UK. Brain.

[2] Sinha S, Satischandra P (2007). Epilepsia partialis continua over last 14 years: Experience from a tertiary care center in south India. Epilepsy Res.

[3] Schapira AH, Cock HR (1999). Mitochondrial myopathies and encephalomyopathies. Eur J Clin Invest.

[4] Gropman AL (2004). The neurological presentations of childhood and adult mitochondrial disease: Established syndromes and phenotypic variations. Mitochondrion.

[5] Schomer DL (1993). Focal status epilepticus and epilepsia partialis continua in adults and children. Epilepsia.

[6] Hori A, Yoshioka A, Kataoka S, Furui K, Tsukada K, Kosoegawa H (1989). Epileptic seizures in a patient with mitochondrial myopathy, encephalopathy, lactic acidosis and stroke like episodes (MELAS). Jpn J Psychiatry Neurol.

[7] Veggiotti P, Colamaria V, Dalla Bernardina B, Martelli A, Mangione D, Lanzi G (1995). Epilepsia partialis continua in a case of MELAS: Clinical and neurophysiological study. Neurophysiol Clin.

[8] Feddersen B, Bender A, Arnold S, Klopstock T, Noachtar S (2003). Aggressive confusional state as a clinical manifestation of status epilepticus in MELAS. Neurology.

[9] Leff AP, Mcnabb AW, Hanna MG, Clarke CR, Larner AJ (1998). Complex partial status epilepticus in late-onset MELAS. Epilepsia.

[10] Corda D, Rosati G, Deiana GA, Sechi G (2006). “Erratic” complex partial status epilepticus as a presenting feature of MELAS. Epilepsy Behav.

[11] Stenqvist L, Paetau A, Valanne L, Suomalainen A, Pihko H (2005). A juvenile case of MELAS with T3271C mitochondrial DNA mutation. Pediatr Res.

[12] Ribacoba R, Salas-Puig J, Gonzalez C, Astudillo A (2006). Characteristics of status epilepticus in MELAS. Analysis of four cases. Neurologia.

[13] Yapici Z, Dincer A, Eraksoy M (2005). Proton spectroscopic findings in children with epilepsy owing to tuberous sclerosis complex. J Child Neurol.

[14] Kim HS, Kim DI, Lee BI, Jeong EK, Choi C, Lee JD (2001). Diffusion-weighted image and MR spectroscopic analysis of a case of MELAS with repeated attacks. Yonsei Med J.

[15] Miyamoto A, Oki J, Takahashi S, Itoh J, Kusunoki Y, Cho K (1997). Serial imaging in MELAS. Neuroradiology.

[16] Pohlmann-Eden B, Hoch DB, Cochius JI, Chiappa KH (1996). Periodic lateralized epileptiform discharges: A critical review. J Clin Neurophysiol.

